# Waterpipe Smoke Exposure Triggers Lung Injury and Functional Decline in Mice: Protective Effect of Gum Arabic

**DOI:** 10.1155/2019/8526083

**Published:** 2019-04-21

**Authors:** Abderrahim Nemmar, Suhail Al-Salam, Sumaya Beegam, Priya Yuvaraju, Badreldin H. Ali

**Affiliations:** ^1^Department of Physiology, College of Medicine and Health Sciences, United Arab Emirates University, P.O. Box 17666, Al Ain, UAE; ^2^Department of Pathology, College of Medicine and Health Sciences, United Arab Emirates University, P.O. Box 17666, Al Ain, UAE; ^3^Department of Pharmacology and Clinical Pharmacy, College of Medicine & Health Sciences, Sultan Qaboos University, P O Box 35, Muscat, 123 Al-Khod, Oman

## Abstract

The prevalence of waterpipe (shisha) tobacco smoking has recently seen a substantial increase worldwide and is becoming a public health problem. Both human and animal studies have established that waterpipe smoke (WPS) increases airway reactivity and inflammation. Gum Arabic (GA) is a prebiotic agent that possesses antioxidant and anti-inflammatory properties. However, its effects on lung toxicity induced by WPS exposure are unknown. Thus, the aim of this study was to investigate the possible salutary effects and underlying mechanisms of GA on WPS-induced pulmonary pathophysiologic effects. C57BL/6 mice were exposed to air or WPS (30 minutes/day for one month) with or without GA treatment in drinking water (15%, *w*/*v*). Exposure to WPS induced an influx of neutrophil polymorphs in the peribronchiolar and interstitial spaces and an increase of tumor necrosis factor-*α* and 8-isoprostane, a marker of lipid peroxidation, concentrations in lung homogenates. The latter effects were significantly mitigated by GA treatment. Likewise, the lung DNA damage induced by WPS exposure was prevented by GA administration. Western blot analysis of the lung showed that GA inhibited nuclear factor kappa-B (NF-*κ*B) expression caused by WPS and augmented that of nuclear factor erythroid 2-related factor 2 (Nrf2). Similarly, immunohistochemical analysis of bronchial epithelial cells and alveolar cells showed a parallel and significant increase in the nuclear expression of Nrf2 and cytoplasmic expression of glutathione in mice treated with GA and exposed to WPS. Moreover, GA administration has significantly prevented airway hyperreactivity to methacholine induced by WPS. We conclude that GA administration significantly declined the physiological, histological, biochemical, and molecular indices of lung toxicity caused by WPS exposure, indicating its beneficial respiratory impact. Considering that GA is a safe agent with health benefits in humans, our data suggest its potential usage in waterpipe smokers.

## 1. Introduction

Waterpipe tobacco smoking (also known as Hubble bubble, shisha, hookah, or narghile) is nowadays the 2^nd^ most common worldwide mean for the consumption of tobacco [1]. Both short-term waterpipe smoking and long-term waterpipe smoking have been reported to cause alteration in pulmonary function [2, 3]. A recent study has demonstrated that weekly waterpipe smoking causes an increase in airway reactivity to mannitol to a similar extent as cigarette smoking (CS) [4]. Additionally, association between WPS and chronic obstructive pulmonary disease has been reported after adjusting for possible confounders such as age and CS [2, 3]. We recently demonstrated that nose-only waterpipe smoke (WPS) exposure increased airway resistance, inflammation, and oxidative stress in mice [5, 6].

Waterpipe smoking is perceived by many as being less addictive and easier to quit than CS. In fact, several waterpipe users reported that one of their reasons for preference of waterpipe smoking over CS is that they are confident of their ability to quit when they want to [7, 8]. However, studies have shown that waterpipe smoking has a similar pattern of addiction as CS and quit rates are very low, with only 28% of ever users having quit [7, 9, 10]. It is clear that permanent cessation of smoking is the ultimate solution for waterpipe smoking and dependency. Nonetheless, in the light of the complexity of the tobacco-dependence syndrome and the factors that contribute to WPS consumption, we believe that any method aiming at alleviating the respiratory toxicity of WPS is of clinical and public health relevance. Consequently, the pursuit for safe, effective, and easily accessible agents that can ameliorate the pathophysiologic effects of WPS is relevant and much needed.

Gum Arabic (GA) is an edible, dried, sticky exudate from *Acacia seyal* and *Acacia senegal* trees, which is rich in soluble dietary fibre [11]. It is commonly used in food manufacturing and pharmaceutical preparations as preservative and emulsifier [11]. Oral intake of GA has been shown to provide several health benefits, such as prebiotic effects in healthy subjects [12, 13]. GA has been demonstrated to exert protective antioxidant and anti-inflammatory actions in animal models of renal failure and cardiac injury [11, 14]. However, the possible protective effect of GA on WPS-induced respiratory toxicity has never been reported, as far as we are aware. Therefore, the present study is aimed at determining whether, and to what extent, would treatment with GA attenuate WPS-induced pulmonary toxicity and at elucidating the possible underlying mechanism. The latter experimental approach would answer to the question as to whether concomitant administration of a safe natural antioxidant and anti-inflammatory prebiotic would mitigate the adverse pulmonary effects of WPS in mice and offer a possible intervention in waterpipe smokers who are unable to quit smoking.

## 2. Material and Methods

### 2.1. Animals and Treatments

This project was appraised and approved by the Institutional Animal Care and Research Advisory Committee of the United Arab Emirates University.

### 2.2. Exposure to WPS

C57BL/6 mice of both genders (8 weeks; United Arab Emirates University, College of Medicine and Health Sciences, animal house) were maintained in a conventional animal house and kept on a 12-hour light-dark cycle (lights on at 6 : 00 am). The animals were put in cages and provided with pelleted food and water ad libitum. After 7 days of adjustment, animals were indiscriminately segregated into 4 different groups, air, WPS, GA plus air, and GA plus WPS. The numbers of male and female animals were similar in the four aforementioned groups.

Mice were put in soft restraints and attached to the exposure tower [5, 6, 15–19]. Using a nose-only exposure system connected to a waterpipe device, the animals were exposed to either air or WPS by their noses (inExpose System, SCIREQ, Canada). Mice were exposed to apple-flavoured tobacco (Al Fakher Tobacco Trading, Ajman, United Arab Emirates). The exposure procedure is controlled by a computerized system (inExpose System, SCIREQ, Canada). A computer-controlled puff was generated each min, producing a 2 s puff duration of WPS exposure followed by 58 s of air. The exposure session lasted 30 min/day [5, 6, 15, 16]. The latter session duration was chosen from the study of Hakim et al. [20] who assessed the respiratory and cardiovascular impact of WPS in healthy subjects. Animals were exposed for one month either to air or to WPS with or without GA (Sigma, St. Louis, MO, USA) treatment which was added to drinking water at a concentration of 15% (*w*/*v*). The dose of GA has been chosen from our prior studies, which demonstrated its efficacy in mitigating chronic renal failure induced by adenine administration in mice and the cardiotoxicity and the disturbance of coagulation caused by WPS exposure in mice [19, 21]. GA has been shown to contain 39–42% galactose, 24–27% arabinose, 12–16% rhamnose, 15–16% glucuronic acid, 1.5–2.6% protein, 0.22–0.39% nitrogen, and 12.5–16.0% moisture [11]. The one-month time point used in the present work has been chosen from our previous work which demonstrated that WPS increased airway resistance, inflammation, and oxidative stress in mice [5].

### 2.3. Histopathology

At the end of the one-month exposure period to either WPS or air with or without GA administration, mice from the various groups were sacrificed by an overdose of sodium pentobarbital and their lungs were removed, washed with ice-cold saline, blotted with filter paper, and weighed. Each lung was dissected, casseted, and fixed in neutral formalin at a concentration of 10%, for a period of 24 hours. After that, the lungs were dehydrated in increasing concentrations of ethanol, cleared with xylene, and embedded with paraffin. From paraffin blocks, sections of 3 *μ*m were prepared and stained with hematoxylin and eosin [22–25]. The latter sections were examined under light microscopy.

### 2.4. Quantification of Tumor Necrosis Factor-*α* (TNF-*α*) and 8-Isoprostane

At the end of the one-month exposure to either WPS or air with or without GA treatment, animals were sacrificed by an overdose of sodium pentobarbital and their lungs were quickly collected and rinsed with ice-cold PBS (pH 7.4) and then snap frozen immediately with liquid nitrogen and stored at -80°C. Later, the tissues were weighed and homogenized with lysis buffer (pH 8) containing NaCl (140 mM), KCl (300 mM), Trizma base (10 mM), EDTA (1 mM), Triton X-100 0.5% (*v*/*v*), sodium deoxycholate 0.5% (*w*/*v*), protease, and phosphatase inhibitor, as described before [22]. The homogenates were centrifuged for 10 min at 3000 × *g* to remove cellular debris, and the supernatants were used for further analysis [22]. Protein content was measured by Bradford's method. The concentrations of TNF-*α* (DuoSet, R&D Systems, Minneapolis, MN, USA) and 8-isoprostane (Cayman Chemical, Michigan, USA) were determined using ELISA Kits [24].

### 2.5. Assessment of DNA Damage by COMET Assay

In separate animals, the lungs of mice obtained at the end of the one-month exposure period to either WPS or air with or without GA treatment were used to assess the DNA damage by COMET assay. The latter was performed as reported before [6, 26, 27], and the measurement of the length of the DNA migration (i.e., diameter of the nucleus plus migrated DNA) was calculated with the image analysis AxioVision 3.1 software (Carl Zeiss, Canada) [6, 28].

### 2.6. Western Blot Analysis for the Quantification of Nuclear Factor Kappa-B (NF-*κ*B) and Nuclear Factor Erythroid 2-Related Factor 2 (Nrf2) Expressions

Protein expressions for NF-*κ*B p65 and Nrf2 were measured on lung tissues harvested from the mice exposed for one month to either WPS or air with or without GA treatment using Western blotting techniques as described before [23, 25].

### 2.7. Immunohistochemical Analysis for the Detection of Nrf2 and Glutathione (GSH)

For immunohistochemistry, sections of lungs (5 *μ*m) collected from mice exposed for one month to either WPS or air with or without GA treatment were cut, dewaxed with xylene, and rehydrated with graded alcohol. After that, the slides were then put in a citrate buffer solution (0.01 M, pH = 6.0) and pretreatment procedures to unmask the antigens were executed in a water bath for 1 hour. Sections were treated with peroxidase and protein block for 15 min each and then incubated with the primary antibodies anti-GSH (rabbit polyclonal antibody, Abcam, CA, USA) and antinuclear factor erythroid-derived 2-like 2 (Nrf2) (rabbit polyclonal antibody, Abcam, CA, USA) for one hour [18]. After conjugation with primary antibodies, sections were washed, incubated with Dako REAL™ EnVision™/HRP for 20 minutes at room temperature (Dako, Agilent, USA), and then followed by washing and addition of DAB chromogen (Dako, Agilent, USA). After that, the sections were counter stained with hematoxylin. Applicable positive controls were utilized. For the negative control, the primary antibody was not added to sections and the entire procedure was performed in the same way as reported above.

The immunohistochemical analyses were done on 5 sections from each mouse at 400x magnification in at least 10 different regions for each section. The immunohistochemical staining of the lung tissue for GSH and Nrf2 was examined semiquantitatively according to the percentage of nuclear staining of bronchial and alveolar cells of each section of the lung for Nrf2 and cytoplasmic staining of bronchial, alveolar, and inflammatory cells of each section of the lung for GSH [18, 29].

### 2.8. Airway Reactivity to Methacholine

Airway hyperreactivity responses were examined using a forced oscillation technique (flexiVent, SCIREQ, Montreal, Canada). Airway resistance (*R*) was evaluated after increasing concentration exposures to methacholine. Mice exposed for one month to either WPS or air with or without GA treatment were anesthetized (i.p. pentobarbital, 70 mg/kg). The trachea was exposed, and an 18-gauge metal needle was inserted into the trachea. Animals were connected to a computer-controlled small animal ventilator and quasi-sinusoidally ventilated with a tidal volume of 10 ml/kg at a frequency of 150 breaths/min and a positive end-expiratory pressure of 2 cm H_2_O to achieve a mean lung volume close to that during spontaneous breathing. After measurement of a baseline, each animal was challenged with methacholine aerosol, generated with an in-line nebulizer and administered directly through the ventilator for 5 s, with increasing concentrations (0, 0.625, 2.5, 10, and 40 mg/ml). *R* was assessed by a “snapshot” protocol each 20 s for 2 min. The mean of these six values was used for each methacholine concentration, unless the coefficient of determination of a measurement was smaller than 0.95. For each animal, *R* was plotted against methacholine concentration (from 0 to 40 mg/ml) [6, 30, 31]. From the latter resistance MCh dose-response curve, an index of airway responsiveness was calculated as the slope of the linear regression using 0–40 mg/ml concentrations, as previously described [5, 23].

### 2.9. Statistics

All statistical analyses were achieved with GraphPad Prism Software version 5. The Shapiro-Wilk normality test was first applied, and the data was found normally distributed. Comparisons between groups were performed by one-way analysis of variance (ANOVA), followed by Newman-Keuls multiple range tests. All the data in figures were reported as mean ± SEM. *P* values < 0.05 are considered significant.

## 3. Results

### 3.1. Lung Histopathology

**Figure 1:**
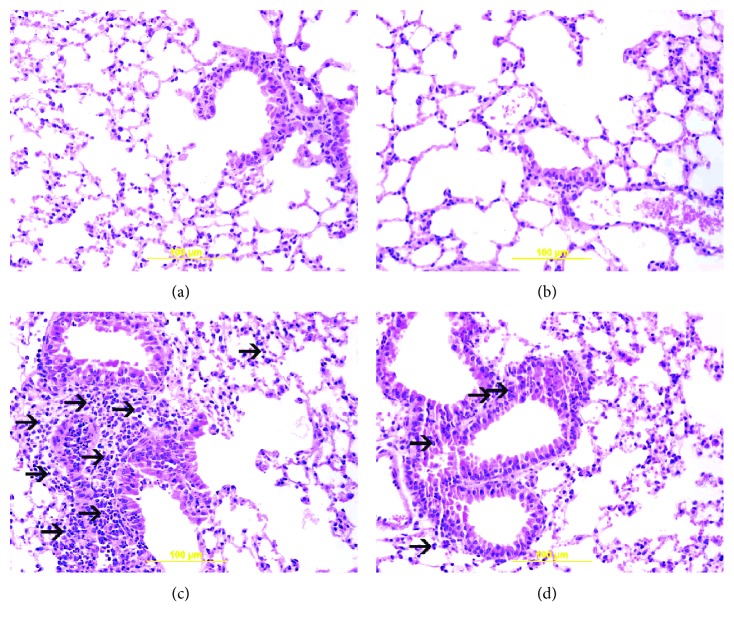
Representative light microscopy sections of lung tissues of mice, at the end of the one-month exposure period to either air (control) or waterpipe smoke (WPS) with or without gum Arabic (GA) administration (15% *w*/*v* in drinking water). (a) The air-exposed group shows normal lung tissue with unremarkable bronchial and alveolar spaces. (b) The GA + air group displays normal lung tissue with unremarkable bronchial and alveolar spaces. (c) The WPS group shows influx of inflammatory cells in the peribronchial and interstitial spaces consisting predominantly of neutrophil polymorphs (arrow). (d) The GA + WPS group displays unremarkable distal airway spaces with mild inflammatory cells in the peribronchial and interstitial spaces consisting predominantly of neutrophil polymorphs (arrow).

Figure 1 shows light microscopy analysis of lung sections obtained from mice exposed to air or WPS with or without GA administration.

The examination of H&E-stained lung sections obtained from the air (Figure 1(a)) and GA + air (Figure 1(b)) groups showed unremarkable morphologic changes. Compared with those of the air-exposed group, lung sections of the WPS-exposed group revealed the presence of a substantial increase in inflammatory cell infiltration in the peribronchiolar and interstitial spaces, formed predominantly out of neutrophil polymorphs (Figure 1(c)). Treatment with GA induced a substantial reduction in the inflammatory cell infiltration (Figure 1(d)).

### 3.2. TNF-*α* and 8-Isoprostane Concentrations in Lung Homogenate

**Figure 2:**
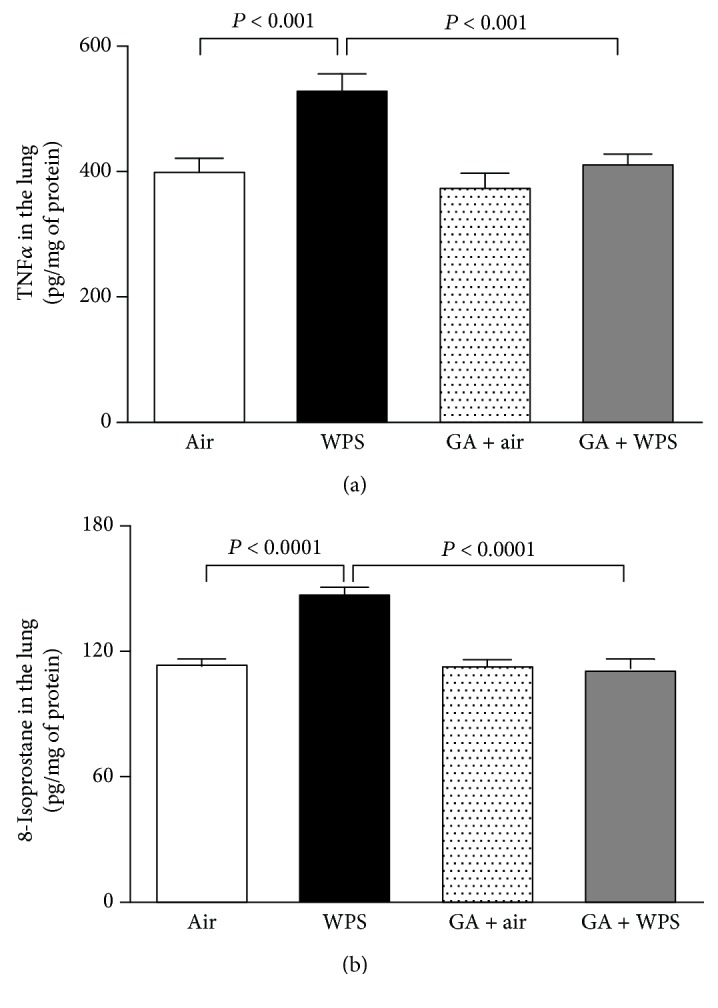
Tumor necrosis factor-*α* (TNF-*α*) (a) and 8-isoprostane (b) concentrations in lung homogenate, at the end of the one-month exposure period to either air (control) or waterpipe smoke (WPS) with or without gum Arabic (GA) administration (15% *w*/*v* in drinking water). Data are mean ± SEM (*n* = 6-8 in each group).

Compared with air-exposed group, WPS exposure caused a significant augmentation in the TNF-*α* concentration in lung homogenates (*P* < 0.001; Figure 2(a)). Following the administration of GA, there was significant inhibition of the increase of TNF-*α* concentrations induced by WPS exposure (*P* < 0.001; Figure 2(a)).

Similarly, 8-isoprostane concentrations were significantly increased by the one-month exposure to WPS compared with those of the air-exposed group (*P* < 0.0001; Figure 2(b)). The latter augmentation of 8-isoprosatne concentrations was significantly prevented in the GA + WPS group (*P* < 0.0001; Figure 2(b)).

### 3.3. Lung DNA Damage

**Figure 3:**
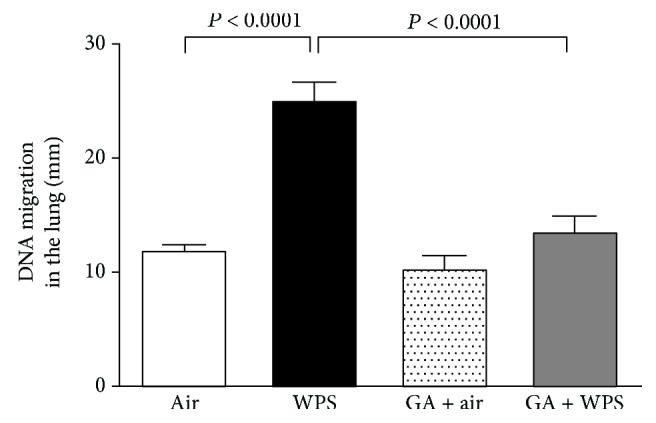
DNA migration (mm) in the lung tissues evaluated by Comet assay, at the end of the one-month exposure period to either air (control) or waterpipe smoke (WPS) with or without gum Arabic (GA) administration (15% *w*/*v* in drinking water). Data are mean ± SEM (*n* = 5 in each group).

Figure 3 illustrates the evaluation of lung DNA damage by Comet assay after one-month exposure to WPS or air, with or without GA administration.

Compared with the air-exposed group, WPS exposure induced a significant increase in DNA migration (*P* < 0.0001; Figure 3). The latter effect was potently averted in the GA + WPS group compared with the WPS group (*P* < 0.0001).

### 3.4. Western Blot Analysis for the Detection of NF-*κ*B

**Figure 4:**
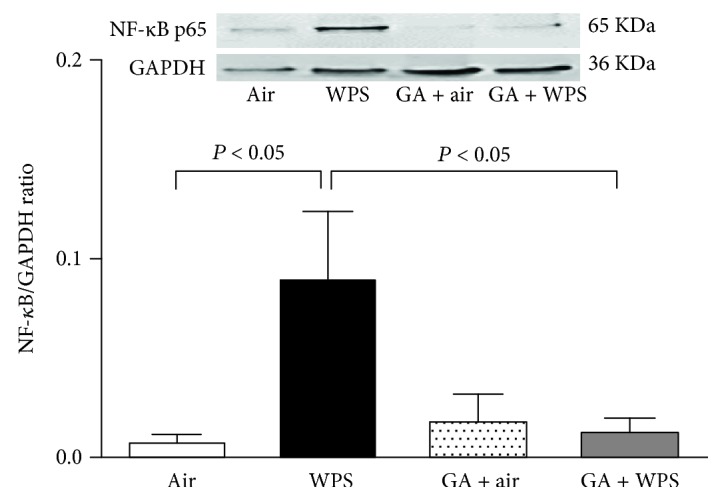
Western blot analysis and graphic representation of nuclear factor kappa-B (NF-*κ*B) protein levels in the lung tissues, at the end of the one-month exposure period to either air (control) or waterpipe smoke (WPS) with or without gum Arabic (GA) administration (15% *w*/*v* in drinking water). Data are mean ± SEM (*n* = 6 in each group).

Figure 4 shows that exposure of mice for one month induced a significant enhancement of NF-*κ*B expression in the lung compared with the air-exposed group (*P* < 0.05). The administration of GA has completely abolished the increase of expression of NF-*κ*B induced by WPS (*P* < 0.05).

### 3.5. Western Blot Analysis for the Detection of Nrf2

**Figure 5:**
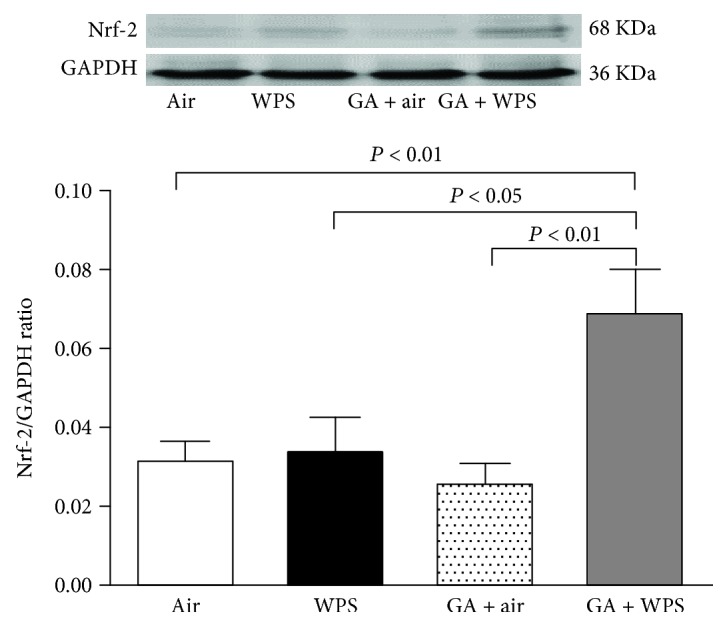
Western blot analysis and graphic representation of nuclear factor erythroid 2-related factor 2 (Nrf2) protein levels in the lung tissues, at the end of the one-month exposure period to either air (control) or waterpipe smoke (WPS) with or without gum Arabic (GA) administration (15% *w*/*v* in drinking water). Data are mean ± SEM (*n* = 5-6 in each group).

Compared with the control group, WPS exposure caused a slight and insignificant increase in Nrf2 expression in the lung (Figure 5). However, following the administration of GA, there was a clear and significant increase in Nrf2 expression in the lung of mice exposed to WPS compared with the WPS alone (*P* < 0.05) or GA + air groups (*P* < 0.01).

### 3.6. Immunohistochemical Analysis for the Detection of Nrf2 and GSH

**Figure 6:**
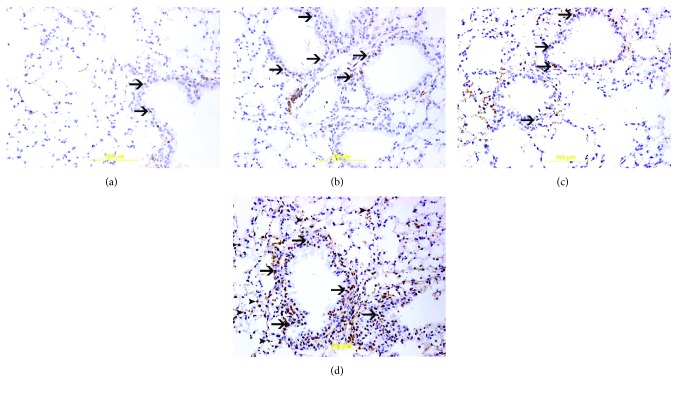
(a–d) Immunohistochemical analysis of the lung tissue sections for the detection of nuclear factor erythroid-derived 2-like 2 (Nrf2) in mice, at the end of the one-month exposure period to either air (control) or waterpipe smoke (WPS) with or without gum Arabic (GA) administration (15% *w*/*v* in drinking water). (a) Representative section of the lung of air-exposed mice showing focal mild nuclear expression of Nrf2 by bronchial epithelium (arrow). (b) Representative section of the lung of the GA + air group displaying nuclear expression of Nrf2 by bronchial epithelium (arrow). (c) Representative section of the lung of WPS-exposed mice showing nuclear expression of Nrf2 by bronchial epithelium (arrow). (d) Representative section of the lung of GA + WPS-exposed mice displaying substantially more nuclear expression of Nrf2 by bronchial epithelium (arrow) and alveolar cells (arrowhead).

There were nuclear and cytoplasmic expressions of Nrf2 by bronchial epithelial cells and alveolar cells in lung sections of all the studied groups but with different intensities and distributions (Figure 6). The nuclear expression of Nrf2 correlates with its antioxidant transcriptional activity while the cytoplasmic expression of Nrf2 does not count for its transcriptional activity. The air-exposed group (14.2 ± 1.8%, *n* = 6) showed mild nuclear expression of Nrf2 by a few bronchial epithelial cells (Figure 6(a)). The GA + air group (29.7 ± 2.0%, *n* = 6) displayed an increase (*P* < 0.0001) in the nuclear expression of Nrf2 by bronchial epithelial cells when compared with the air-treated group (Figure 6(b)). The WPS-exposed group (34.7 ± 2.5, *n* = 6) showed an increase (*P* < 0.0001) in the nuclear expression of Nrf2 by bronchial epithelial cells when compared to the air-exposed group (Figure 6(c)). When compared with the WPS-exposed group, the GA + WPS group (77.1 ± 2.5, *n* = 6) displayed a considerable increase (*P* < 0.0001) in the nuclear expression of Nrf2 by bronchial epithelial cells and alveolar cells (Figure 6(d)).

**Figure 7:**
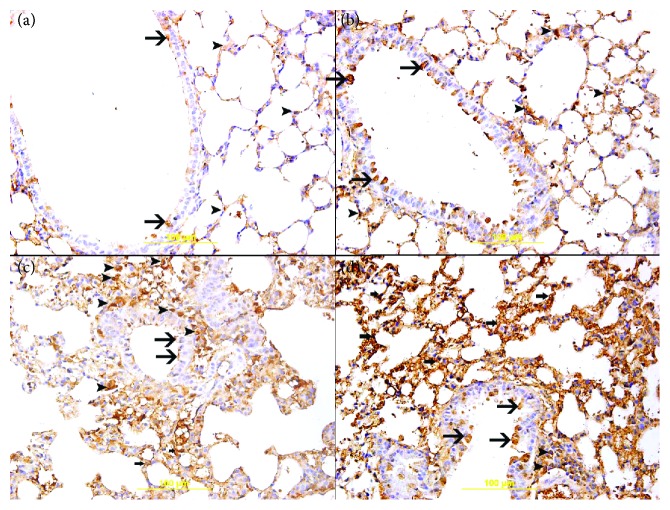
(a–d) Immunohistochemical analysis of the lung tissue sections for the detection of glutathione (GSH) in mice, at the end of the one-month exposure period to either air (control) or waterpipe smoke (WPS) with or without gum Arabic (GA) administration (15% *w*/*v* in drinking water). (a) Representative section of the lung of air-exposed mice showing mild cytoplasmic expression of GSH by a few bronchial epithelial cells (arrow) and alveolar cells (arrowhead). (b) Representative section of the lung of the GA + air group showing increase in the cytoplasmic expression of GSH by bronchial epithelial cells (arrow) and alveolar cells (arrowhead) when compared with the air-exposed group. (c) Representative section of the lung of WPS-exposed mice showing increase in the cytoplasmic expression of GSH by bronchial epithelial cells (thin arrow), alveolar cells (thick arrow), and inflammatory cells (arrowhead) when compared with that of the air-exposed group. (d) Representative section of the lung of GA + WPS-exposed mice showing substantially more increase in the cytoplasmic expression of GSH by bronchial epithelial cells (thin arrow), alveolar cells (thick arrow), and a few inflammatory cells (arrowhead) when compared with the WPS-exposed group or GA + air group or air group.

Overall, there was cytoplasmic expression of GSH by bronchial epithelial cells, alveolar cells, and inflammatory cells in lung sections of the studied groups but with different intensities and distributions (Figure 7). The air-exposed group (15.7 ± 2.1, *n* = 6) showed mild cytoplasmic expression of GSH by a few bronchial epithelial cells and alveolar cells (Figure 7(a)). The GA + air group (32.8 ± 3.8, *n* = 6) displayed an increase (*P* < 0.01) in the cytoplasmic expression of GSH by bronchial epithelial cells and alveolar cells when compared with the air-exposed group (Figure 7(b)). The WPS-exposed group (54.3 ± 5.2, *n* = 6) showed an increase (*P* < 0.0001) in the cytoplasmic expression of GSH by bronchial epithelial cells, inflammatory cells, and alveolar cells when compared with the air-treated group (Figure 7(c)). The GA + WPS group (80.4 ± 2.9, *n* = 6) displayed a significant increase (*P* < 0.0001) in the cytoplasmic expression of GSH by bronchial epithelial cells and alveolar cells when compared with the WPS-exposed groups (Figure 7(d)).

### 3.7. Airway Hyperreactivity to Methacholine

**Figure 8:**
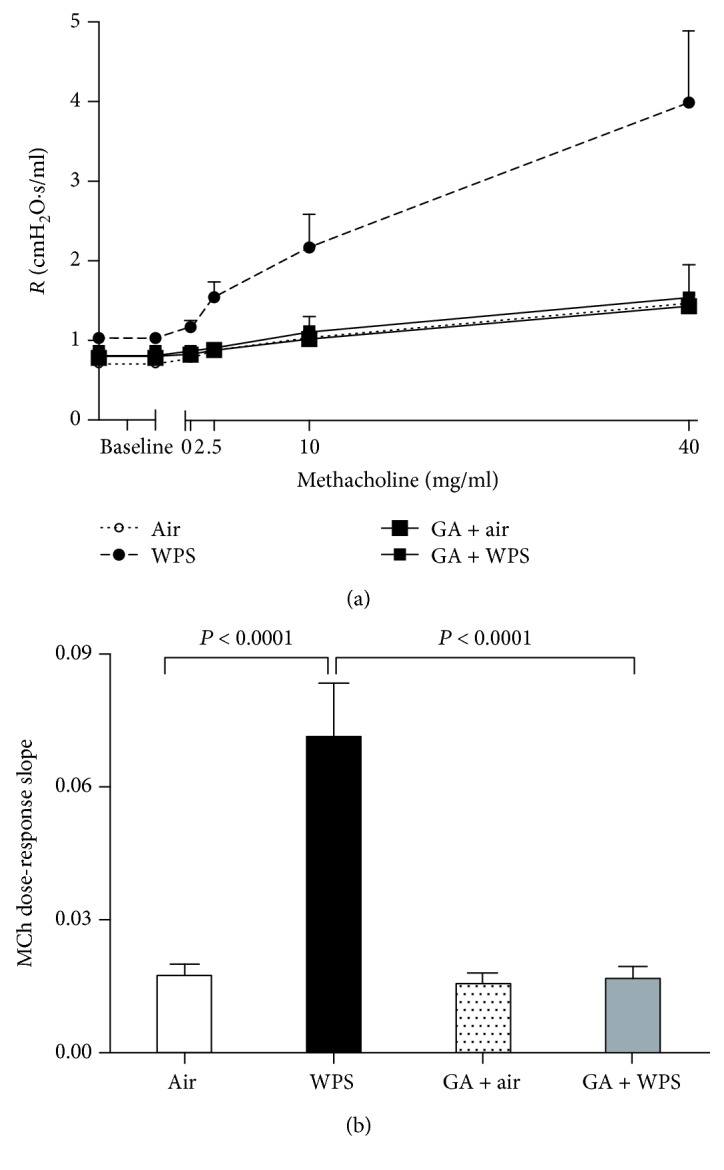
Airway hyperresponsiveness. The airway resistance (*R*), after increasing concentrations of methacholine (MCh) (0–40 mg/ml), was measured via the forced oscillation technique (flexiVent), at the end of the one-month exposure period to either air (control) or waterpipe smoke (WPS) with or without GA administration (15% *w*/*v* in drinking water). Dose-response relationship of total respiratory system resistance to increasing doses of MCh (a). From the resistance MCh dose-response curve in (a), an index of airway responsiveness was calculated as the slope of the linear regression using 0–40 mg/ml concentrations (b). Data are mean ± SEM (*n* = 8-9).

Figure 8 illustrates the airway resistance measured by forced oscillation technique after increasing concentrations of methacholine (0-40 mg/ml) in the air, WPS, GA + air, and GA + WPS groups. Compared with the air-exposed group, airway resistance was significantly and dose-dependently increased in WPS-exposed mice. No difference in airway resistance has been seen between the air and GA + air groups. However, GA administration has completely prevented the increase of airway resistance triggered by WPS (Figure 8(a)).

From the airway resistance methacholine dose-response curve, an index of airway reactivity was calculated as the slope of the linear regression using 0–40 mg/ml concentration (Figure 8(b)). Compared with the air-exposed group, the methacholine dose-response slope was significantly augmented in the WPS-exposed group (*P* < 0.0001). The latter effect was significantly abrogated by GA treatment (*P* < 0.0001).

## 4. Discussion

The present study provided experimental evidence that GA administration exerts significant protective effects against WPS-induced respiratory pathophysiological effects. We demonstrated that GA prevented pulmonary inflammation, oxidative stress, DNA damage, and restored the impairment of lung function. We also found that the combination of GA with WPS exposure prevented the expression of NF-*κ*B and induced an overexpression of Nrf2.

Waterpipe tobacco smoking contains substantial amounts of toxic compounds including nicotine, tar, CO, heavy metals, and volatile aldehydes [32, 33]. Compared with CS, it has been previously demonstrated that regular smoking of waterpipe leads to analogous concentrations of nicotine absorption and greater levels of CO [32, 33]. Experimental studies aiming at investigating the impact of WPS or CS exposure currently use two major exposure systems, which comprise nose-only and whole-body systems [17, 34–36]. Compared with the whole-body exposure system, the nose-only exposure model used presently is better controllable and more accurate because it circumvents the shortcoming related to ingestion of nicotine, tar, or other substances when the mice or rats clean their fur. The nose-only exposure system best resembles the human situation [34, 35]. Similarly to our previous studies, in the current study, the exposure session to WPS lasted 30 min [15, 17]. The latter is comparable to the exposure session reported in the human study [20]. Moreover, we have recently reported that the levels of carboxyhemoglobin found in mice exposed to nose-only WPS were comparable with those reported in waterpipe smokers [17].

Our data show that GA substantially averted WPS exposure-induced pulmonary inflammation and oxidative stress by alleviating neutrophil infiltration in the peribronchiolar and interstitial spaces and reducing the concentrations of the proinflammatory cytokine TNF-*α* and 8-iso-prostane, a marker of lipid peroxidation which is produced by the peroxidation of arachidonic acid, catalyzed by free radicals [37]. GA has been reported to exert ameliorative effects on adenine-induced chronic renal disease in rats and mice and in potassium bromate-induced kidney injury in rats by a mechanism involving inflammation and oxidative stress [21, 38]. We have recently shown that exercise training mitigated subchronic WPS-induced airway inflammation and oxidative stress [23]. A growing body of evidence suggests that inflammation and oxidant-antioxidant imbalance could exert a major role in the initiation and development of lung injury. As a result of oxidant-antioxidant imbalance, the increase in the levels of reactive oxygen and nitrogen species could cause cell membrane injury and DNA damage [39]. Our data show that along with inflammation and oxidative stress, WPS exposure induced DNA damage in the lung assessed by Comet assay and that the treatment with GA prevented this toxic effect. The latter protective effect of GA could be explained by its established anti-inflammatory and antioxidant actions [40]. GA has also been shown to prevent DNA damage in adenine-induced chronic kidney disease in rats [41].

To further investigate the mechanisms whereby GA exerts its beneficial action, we have evaluated the expression of the nuclear transcription factor, NF-*κ*B, which has been shown to play a critical role in the regulation of the pathological process involving inflammation and oxidative stress in various organs including the lung [42]. Here, we show that WPS exposure for one month caused a significant activation of NF-*κ*B and that the administration of GA has abrogated this effect. GA has been associated with modulation of NF-*κ*B and reduction of the inflammatory response in an animal model of cathartic-induced intestinal dysfunction [43]. Moreover, it was shown that GA alone or in combination with selenium-enriched yeast exerts a protective effect against CCl_4_-induced hepatotoxicity through inhibition of NF-*κ*B and TNF-*α* [44].

Nrf2 is a pervasive master transcription factor, which controls antioxidant response element-induced expressions of antioxidant enzyme and cytoprotective proteins [45]. Nrf2 is recognized to be activated by phosphorylation change through various protein kinase pathways leading to Keap1 and Nrf2 dissociation and nuclear Nrf2 translocation and controls antioxidant response element reaction [45]. Nrf2 agonists, such as phytochemical isothiocyanates and synthetic triterpenoids, were successfully applied experimentally in animal models of oxidant-induced pulmonary injury [45]. To verify the possible contribution of Nrf2 in the protective effect of GA against WPS-induced lung toxicity, we performed Western blot and immunohistochemical analysis of the lung. Our data show that one-month exposure to WPS induced an insignificant increase of Nrf2 expression. However, the combination of GA with WPS exposure triggered a considerable increase in Nrf2 expression by Western blot analysis. Likewise, using immunohistochemistry, we found a clear increase in the nuclear expression of Nrf2 by bronchial epithelial cells and alveolar cells in the GA + WPS group compared with the WPS group, confirming the activation of this transcription factor. In conjunction with Nrf2 activation in the GA + WPS group, we found in the same group a significant increase in the expression of the antioxidant GSH by bronchial epithelial cells and alveolar cells. Our data suggests that GA exerts a protective effect against WPS-induced pulmonary toxicity by preventing inflammation and oxidative stress via the activation of the Nrf2 signalling pathway, which increases the production of antioxidants such as GSH. The latter result suggests that GA may be considered as an Nrf2 inducer. This finding is novel as the effect of GA on Nrf2 expression has never been reported before. It has been recently reported that exercise training mitigated WPS-induced lung injury by activating Nrf2 signalling pathways [23]. Also, it has been demonstrated that platycodin D, a natural agent with anti-inflammatory properties, alleviates the injurious effect of CS exposure by inhibiting inflammation and oxidative stress by activating Nrf2 expression [46].

A recent study in humans has reported that waterpipe smoking caused an increase in airway reactivity to mannitol to a similar extent as CS [4]. We have also provided experimental evidence that WPS in mice causes airway hyperresponsiveness to methacholine and that exercise training mitigates this effect [5, 6, 23]. However, the effect of GA on airway resistance has not been documented before. Accordingly, we have evaluated the in vivo efficacy of GA on WPS-induced alteration in airway hyperreactivity responses. Our data showed that, as expected, WPS exposure for one month induced an increase in airway reactivity, and it also showed, for the first time, that GA has effectively prevented the increase of airway hyperreactivity induced by WPS, confirming the ability of GA to protect the respiratory system from the injurious effect of WPS.

The possible useful effects of GA on microbiota and the identification of the composition of the gut microbial community in rodents receiving different doses of GA remain to be established. There is a study showing that GA has beneficial effect of inflammatory conditions in the gut [47] and, in general, dietary fibres (like GA) are established to have a beneficial action on health and against diseases involving inflammation and oxidative stress [48].

Taken together, our data show that one-month exposure to WPS induces lung inflammation, oxidative stress, DNA injury, and increase in airway reactivity and that concomitant GA treatment alleviated these effects through a mechanism involving the inhibition of NF-*κ*B and activating Nrf2 expressions. Additional work is required to assess whether the protective effects of GA would remain constant following chronic exposure to WPS and which ingredient in GA might be responsible for our observed findings. Our experimental findings may set the stage to further controlled clinical studies to assess the clinical effectiveness of GA in current and past waterpipe smokers and set the dose and frequency of use of GA in human subjects.

## Data Availability

The data that support the findings of this study are available from the corresponding author, Abderrahim Nemmar, upon reasonable request.
